# Supplementary material for: Coronary heart disease and stroke mortality trends in Brazil 2000–2018
Source: PLoS One. 2021 Sep 2;16(9):e0253639. doi: 10.1371/journal.pone.0253639 (PMC8412280; doi:10.1371/journal.pone.0253639)
Supplement: Attachment — Submitted filename: REBUTTAL LETTER.docx [file pone.0253639.s001.docx]

18 May 2021

**REBUTTAL LETTER**

PONE-D-21-08855
Coronary Heart Disease and stroke mortality trends in Brazil 2000-2018

PLOS ONE

Dear Venkata Naga Srikanth Garikipati, PhD

We would like to thank you and the reviewers for your time to provide feedback on our paper. We have addressed all reviewers’ comments below and Journal requirements.

**Reviewer #1**

**Comments to the Author:**

“The authors have written the paper technically sound, written in good English, statistical analysis are also well performed. The authors aim to understand the Coronary Heart Disease and stroke mortality trends in Brazil 2000-2018. I personally think are successful in addressing the issue.”

**Response:** Thank you for these constructive comments.

**Reviewer #2**

**Comments to the Author:**

“The manuscript PONE-D-21-08855, titled “Coronary Heart Disease and stroke mortality trends in Brazil 2000-2018” by Patricia et al., they have analysed the trend of mortality due to coronary heart disease and stroke in the adult population in Brazil.

They have reported the decrease in crude mortality rates in both sexes and all age groups with exception in males over 85 years where the rate was increased. The declining trend was observed in males in age group 35 to 44 years and females in 65-74 years. The gross mortality for strokes was also decreased in both sexes and age groups between 2000 and 2018, with no exceptions. But this paper cannot be accepted under Research article category as the data is not sufficient and does not match the standard of PLOS One.”

**Response:** #1. Reviewer 2 is unhappy with data not being enough. We will be grateful if the reviewer can elaborate more and indicate the purposes and how the data is inadequate, we can provide a better response.

#2. The reviewer then states that the manuscript should not be accepted as a Research Article, as it doesn't comply with the journal standards. We politely disagree with the reviewer. For example., our paper describes the use of join point regression analysis on national mortality data in a similar way as we and others discussed trends in other settings (see for example <https://journals.plos.org/plosone/article?id=10.1371/journal.pone.0114027>

or <https://journals.plos.org/plosone/article?id=10.1371/journal.pone.0059608#:~:text=Between%201982%20and%202006%2C%20the,year)%20(Table%201>).

We kindly ask the reviewer to specify which aspects do not fit the criteria of the journal, as it will enable us to address the points more precisely.

**Journal Requirements**

**Comments to the Author:**

1. “Please review your reference list to ensure that it is complete and correct.”

**Response:** Thank you for raising this point. Now the reference list meets PLOS ONE's style requirements.

2. “Please ensure that your manuscript meets PLOS ONE's style requirements, including those for file naming.”

**Response:** The manuscript with track changes was adapted according to: https://journals.plos.org/plosone/s/file?id=wjVg/PLOSOne_formatting_sample_main_body.pdf

and

[https://journals.plos.org/plosone/s/file?id=ba62/PLOSOne_formatting_sample_title_authors_affiliations.pdf](about:blank)

3. “Please provide additional details regarding participant consent. In the ethics statement in the Methods and online submission information, please ensure that you have specified (1) whether consent was informed and (2) what type you obtained (for instance, written or verbal, and if verbal, how it was documented and witnessed). If your study included minors, state whether you obtained consent from parents or guardians. If the need for consent was waived by the ethics committee, please include this information.

If you are reporting a retrospective study of medical records or archived samples, please ensure that you have discussed whether all data were fully anonymized before you accessed them and/or whether the IRB or ethics committee waived the requirement for informed consent. If patients provided informed written consent to have data from their medical records used in research, please include this information”.

**Response:** Thank you for spotting this omission. Now in the Methods and online submission information reads:

*“The research protocol was approved by The Ethics Committee of Federal University of Paraíba with consent number 3.843.739. As these are secondary data made available on public domain sites of the Brazilian Unified Health System, ethics committee waived the requirement for informed consent.”*

4. “Thank you for stating in your Funding Statement:

 [This work was partially supported by Conselho Nacional de Desenvolvimento
Científico e Tecnológico – CNPq; Grant number 442891/2019-9. Agency that granted a
scholarship and resources for the purchase of equipment.].

Please provide an amended statement that declares *all* the funding or sources of support (whether external or internal to your organization) received during this study, as detailed online in our guide for authors at [http://journals.plos.org/plosone/s/submit-now](about:blank).  Please also include the statement “There was no additional external funding received for this study.” in your updated Funding Statement.

Please include your amended Funding Statement within your cover letter. We will change the online submission form on your behalf.”

**Response:** Thank you for your comment, we have now rewritten.

*“The National Council for Scientific and Technological Development (Conselho Nacional de Desenvolvimento Científico e Tecnológico – CNPq) supported all the funding or sources of support received during this study; Grant number 442891/2019-9. The funders had no role in study design, data collection and analysis, decision to publish, or preparation of the manuscript.”*

The amended Funding Statement was added in our cover letter.

5. “Please upload a new copy of Figures 2 & 3 as the detail is not clear.”

**Response:** Thank you for your suggestion. The figures were uploaded to the Preflight Analysis and Conversion Engine (PACE) digital diagnostic tool and they have been replaced.

Kind regards,

The author

On behalf of the co-authors
